# Characterization of the 1-(5-(4,5-Dimethyl-1,3,2-dioxoborolan-2-yl)thiophen-2-yl)ethanone Using NMR ^13^C, ^1^H and ^11^B through the Density Functional Theory

**DOI:** 10.3390/ma16083037

**Published:** 2023-04-12

**Authors:** Ulises J. Guevara, Jesús B. Núñez R., Rafael Lozada-Yavina, Anton Tiutiunnyk, Laura M. Pérez, Pablo Díaz, Neudo Urdaneta, David Laroze

**Affiliations:** 1 Instituto de Alta Investigación, Universidad de Tarapacá, Arica 1000000, Chile; 2 Departamento de Biología, Universidad Politécnica Territorial del Oeste de Sucre “Clodosbaldo Russian”, Cumaná 6101, Venezuela; 3 Facultad de Ciencias Básicas, Universidad Católica del Maule, Talca 3480112, Chile; 4 Facultad de Ciencias e Ingeniería, Universidad Tecnológica del Perú, Lima 15046, Peru; 5 Departamento de Física, FACI, Universidad de Tarapacá, Arica 1000000, Chile; 6 Departamento de Ciencias Físicas, Universidad de La Frontera, Casilla 54-D, Temuco 4780000, Chile; 7 Departamento de Química, Universidad Simón Bolívar (USB), Caracas 1020, Venezuela

**Keywords:** density functional theory, nuclear magnetic resonance, chemical shielding, chemical shift

## Abstract

The use of computational methods that allow us to perform characterization on new compounds is not a novelty; nevertheless, the degree of complexity of the structures makes their study more challenging since new techniques and methods are required to adjust to the new structural model. The case of nuclear magnetic resonance characterization of boronate esters is fascinating because of its widespread use in materials science. In this paper, we use density functional theory to characterize the structure of the compound 1-[5-(4,5-Dimethyl-1,3,2-dioxaborolan-2-yl)thiophen-2-yl]ethanonea by means of nuclear magnetic resonance. We studied the compound in its solid form with the PBE–GGA and PBEsol–GGA functionals, with a set of plane wave functions and an augmented wave projector, which included gauge in CASTEP and its molecular structure with the B3LYP functional using the package Gaussian 09. In addition, we performed the optimization and calculation of the chemical shifts and isotropic nuclear magnetic resonance shielding of ${}^{1}$H, ${}^{13}$C, and ${}^{11}$B. Finally, we analyzed and compared the theoretical results with experimental diffractometric data observing a good approximation.

## 1. Introduction

The computational models and methods used to obtain the thermodynamic and physical properties of systems with a certain number of particles have evolved so fast that it is feasible to analyze structures with hundreds of atoms in unitary cells, thus resulting in a more approachable task [1,2,3]. However, the solution to these problems leads to new questions, challenges, and materials to study, as well as new applications of these methods and models to different areas such as medicine, chemistry, and materials science [4,5,6,7].

A typical example of applying these models and methods lies in the characterization of new compounds related to boronate esters, where the relation between the atoms and the peaks in magnetic resonance spectroscopy occasionally turns out to be difficult [8,9,10]. In certain chemical reactions, the boronic acid group in some organic compounds sometimes causes the cleavage of the B–C bond, so it is necessary to protect the boronic acid group with a diol [11]. The compounds with boronate esters represent valuable means in organic synthesis, particularly in the Suzuki–Miyaura coupling reaction [12]. They are useful for carbohydrate detection [13] due to their capacity to produce cyclic esters with suitable diols [14]. They show biological activities, such as antidepressant, antiallergic, anesthetic, and anti-Alzheimer agents, as well as proteasome and lipogenic inhibitors [15]. For this reason, when a new molecule appears, it is necessary to know its properties and possible uses.

Crystallographic and spectroscopic studies related to new compounds of boronate remain unexplored to a great extent, and only a few crystalline structures have been reported so far. The main goal of this work is to study the isotropic shielding, ${}^{1}$H, ${}^{13}$C, ${}^{11}$B, NMR chemical shifts of the 1-(5-(4,5-dimethyl-1,3,2-dioxoborolan-2-yl)thiophen-2-yl)ethanone [16] by applying density functional theory. The paper is arranged as follows: In Section 2, the theoretical background is given. In Section 3, the methodology is presented, whereas, in Section 4, the results are presented and analyzed. Finally, the conclusion is given in Section 5.

## 2. Theoretical Background

In this section, we provide the necessary background to understand our computational results that are compared with experimental data.

### 2.1. Nuclear Magnetic Resonance

The Nuclear Magnetic Resonance (NMR) technique provides detailed information about the structure and chemical environment of molecules. A spinning nucleus with a non-zero, positive charge exhibits a nuclear magnetic moment that interacts with an applied magnetic field [17]. When the NMR technique is applied, a chemical compound is added, which will act as the reference to measure the chemical shift. Tetramethylsilane ($Si{\left(CH_{3}\right)}_{4}$) [18] is one of the most used reference compounds in the shielding calculations of ${}^{13}$C and ${}^{1}$H, while Boron trifluoride diethyl etherate ($BF_{3}OEt_{2}$) is one of the most preferred choices for ${}^{11}$B.

### 2.2. Pseudopotentials in a Magnetic Field

The pseudopotential approach originated with the orthogonal plane wave (OPW) method proposed by Herring [19]. Later, an extension was carried out by incorporating additional terms in the potential that leads to the Phillips–Kleinman pseudopotential [20]. The formulation of these pseudopotentials allowed for their subsequent evolution, leading to what is known as norm conservation pseudopotentials, where Topp, Hopfield, Starkloff, Joannopoulos & Hamann [21,22,23] carried out significant advancements concerning the consideration of the equality in the wave functions and pseudo wave functions beyond the nucleus radius. However, a widely used pseudopotential nowadays is the ultra-soft pseudopotential (USPP) introduced by Vanderbilt [24], since it considers lower cutting energy that allows for a smaller number of pseudo-wave functions. Finally, the idea of splitting the spheric crystalline around the atoms and assuming that inside the sphere the potential is harmonic led Blöchl [25] to introduce the projector augmented wave (PAW) method. Later, Pickard and Maury [26] introduced the gauge including projected augmented wave (GIPAW) method, which is a modification of the original PAW method introduced by Blöchl, where an invariant transformation based on the application of a uniform magnetic field is proposed. The GIPAW method allows us to calculate the isotropic shielding σ and the functional density theory-estimated NMR chemical displacements, considering the pseudopotential in a uniform magnetic field.

## 3. Methodology

### 3.1. Analysis and Optimization

In order to calculate the isotropic shielding and the chemical displacement, it is necessary to start with an optimization of the structures. i.e., a state of relaxation of the crystalline structures. Next, optimization is carried out for both the unitary cells of the $C_{10}H_{13}BO_{3}S$ structure as well as the cells that enclose the $Si{\left(CH_{3}\right)}_{4}$ molecules in the case of the ${}^{1}$H and ${}^{13}$C reference shielding and $BF_{3}OEt_{2}$ molecules in the case of the ${}^{11}$B reference shielding, respectively.

### 3.2. Optimization of the $Si{\left(CH_{3}\right)}_{4}$ and $BF_{3}OEt_{2}$ Molecules, and the Unitary Cell of the $C_{10}H_{13}BO_{3}S$ Structure

**Figure 1:**
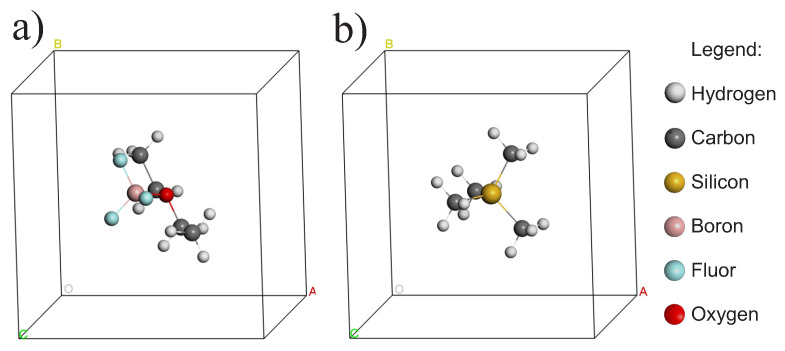
(**a**) 1000 Å${}^{3}$ cell for the $BF_{3}OEt_{2}$ molecule. (**b**) 1000 Å${}^{3}$ cell for the $Si{\left(CH_{3}\right)}_{4}$ molecule. The legend applies to both cells.

By placing the molecule inside a sufficiently large box, we are avoiding the interaction of the molecule with its neighbors in the periodicity, so we build a cell of 1000 Å${}^{3}$ for the molecule $BF_{3}OEt_{2}$ (Figure 1a) and another of 1000 Å${}^{3}$ for the molecule of $Si{\left(CH_{3}\right)}_{4}$ (Figure 1b). In other words, a cell of 10 Å × 10 Å × 10 Å, which isolates molecules from each other.

For the optimization of the molecules $Si{\left(CH_{3}\right)}_{4}$ and $BF_{3}OEt_{2}$ in cells, Perdew–Burke–Ernzerhof (PBE) exchange and correlation functionals were used [27], and Perdew–Burke–Ernzerhof for solids (PBEsol), both with generalized gradient approximation and a plane-wave basis set, over a Γ point of reciprocal space with a cutoff energy of 300 eV, a convergence tolerance parameter of $1.0\times 10^{-5}$ eV/atom, a maximum strength of 0.05 eV/Å, a maximum stress 0.01 GPa, and a maximum displacement of $2.0\times 10^{-4}$ Å, the pseudopotential is one generated at runtime on the server “on the fly,” which differs from the norm-conserving and ultra-soft pseudopotentials in that they use tabulated data for the projections. The algorithm used in the optimization was BFGS.

**Figure 2:**
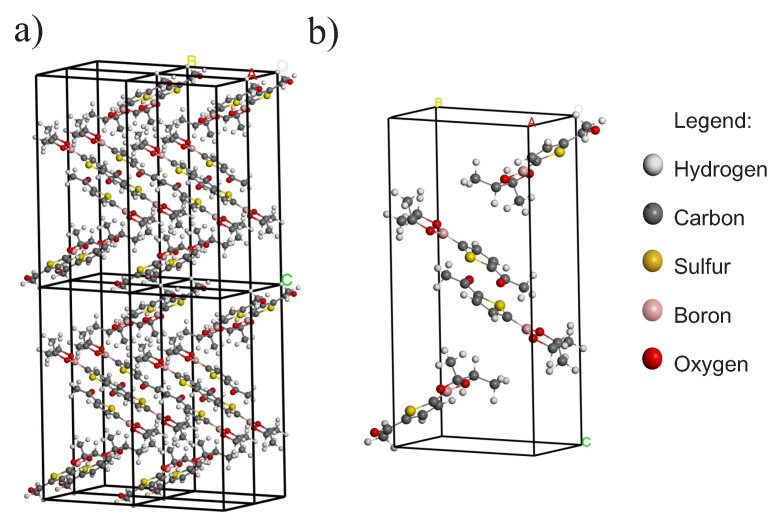
(**a**) Periodic structure. (**b**) Unitary cell of the $C_{10}H_{13}BO_{3}S$ structure.

For the optimization of the unit cell of the structure $C_{10}H_{13}BO_{3}S$ (Figure 2), three cases are considered. The first two use the PBE–GGA and the PBEsol–GGA functionals in the reciprocal space of the periodic structure using CASTEP [27]. The experimental results and lattice parameters obtained with the PBE and the PBEsol are presented in Table 1. The third case with the functional B3LYP on the molecule uses the software Gaussian 09 [28]. We work on a Monkhorst-Pack scheme to generate points k in a lattice 11×6×3, with a cutoff energy of 550 eV and convergence tolerance parameter of $1.0\times 10^{-5}$ eV/atom, such that the maximum strength is $5.0\times 10^{-2}$ eV/Å, maximum stress is $1.0\times 10^{-2}$ GPa, and the maximum displacement is $2.0\times 10^{-4}$ Å. The used psesudopotential was “on the fly”.

The optimizations of the molecules 1-(5-(4,5-dimethyl-1,3,2-dioxaborolan-2-yl)thiophen-2-yl)ethan-1-one, $Si{\left(CH_{3}\right)}_{4}$ and $BF_{3}OEt_{2}$ were made with the package Gaussian 09 [28], and were executed using the base set 6-311+G(2d,p) [29,30,31] and the exchange–correlation functional B3LYP [32,33]. The default algorithm for the minimization is the Berny algorithm using GEDIIS [34]. The Hessian is updated unless an analytic Hessian has been computed or it is the first step, in which case an estimate of the Hessian is made. The convergence is tested against criteria for the maximum force component $4.5\times 10^{-4}$, root-mean-square force $3.0\times 10^{-4}$, maximum step component $1.8\times 10^{-3}$, and root-mean-square step $1.2\times 10^{-3}$. The step is the change between the most recent point and the next to be computed (the sum of the linear and quadratic steps). Let us also comment that using the results of the positions and the optimized lattice of the $C_{10}H_{13}BO_{3}S$ compound, we have performed the calculation of the band structure. The detailed results are given in Appendix A.

### 3.3. NMR of the $Si{\left(CH_{3}\right)}_{4}$ and $BF_{3}OEt_{2}$ Molecules, and the Unitary Cell of the $C_{10}H_{13}BO_{3}S$ Structure

Once the $Si{\left(CH_{3}\right)}_{4}$ and $BF_{3}OEt_{2}$ unitary cells have been relaxed, the pseudopotential used for the magnetic resonance calculation is the same as the wave projector used for relaxation, with a cut-off energy of 250 eV. The integration of the Brillouin zone was defined as a grid with points k in a 4×4×4 lattice in the reciprocal space, and the functionals PBE–GGA, PBEsol–GGA in the case of the periodical structure and B3LYP for the case of the molecule. A self consistent field (SCF) tolerance of $1.0\times 10^{-5}$ eV/atom and a self consistence maximum cycle of 150 cycles. For the unitary cell of the $C_{10}H_{13}BO_{3}S$ structure, a cut-off energy of 550 eV is used. The integration of the Brillouin zone was defined as a grid with points k in a 11×6×3 lattice in the reciprocal space and the functional ones mentioned above. A self consistent field (SCF) tolerance and a self consistence maximum cycle were repeated in this calculation.

### 3.4. Software

CASTEP software was used to optimize the geometric structure and obtain the NMR spectra of the $C_{10}H_{13}BO_{3}S$ compound. Plane–wave basis sets and conserved norm pseudopotential were used to optimize the structures and an “on the fly” pseudopotential to obtain the NMR spectra [26,36,37]. Calculations performed with package Gaussian 09 [28], version D.01, were realized at 298.15 K, 1 atm of pressure and taking into account the effect of the solvent (chloroform).

## 4. Results and Discussion

The structural parameters of the composite boronate were obtained by their geometrical optimization, as shown in Table 2, Table 3, Table 4 and Table 5, in a representative manner. In all Tables, we show the results using the three different used DFT techniques.

The carbon–hydrogen bond lengths in the $C_{10}H_{13}BO_{3}S$ compound exhibit a slight variation after optimization (Table 2). This is due to the higher freedom of motion of the hydrogen atoms, which can be compared with the carbon–carbon bond lengths (Table 3), where the variation is smaller since the carbon atoms have stronger bonds. Table 4 depicts the bond lengths and optimized lengths in the $Si{\left(CH_{3}\right)}_{4}$ compound. The variation, although not significant, is acceptable for the calculations of the reference isotropic shielding; $\sigma_{\mathrm{ref}}=179.400$ ppm for ${}^{13}$C and $\sigma_{\mathrm{ref}}=30.570$ ppm for hydrogen (see Table 6). The calculated values are in agreement with those reported in the literature. Table 5 shows the bond lengths and optimized lengths in the $BF_{3}OEt_{2}$ compound and Table 6 shows the isotropic shielding; $\sigma_{\mathrm{ref}}=88.080$ ppm (reference value isotropic shielding) for ${}^{11}$B.

**Figure 3:**
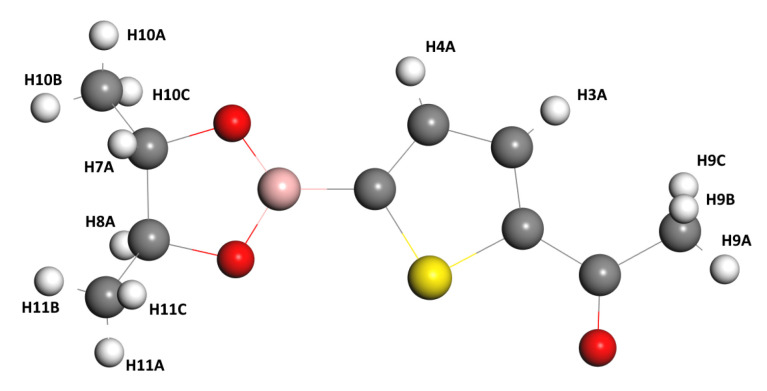
Hydrogen atoms of the $C_{10}H_{13}BO_{3}S$ compound.

Before analyzing the results of the theoretical calculations on NMR, let us comment that all the labels of the Hydrogen atoms are referred to in Figure 3.

**Figure 4:**
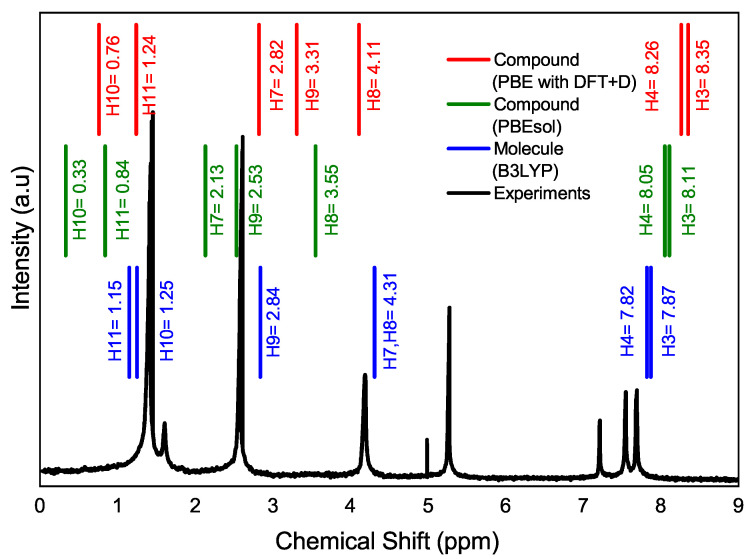
Intensity as a function of the chemical shift. The panels compare the experimental nuclear magnetic resonance in (black line) with different functionalities and in different situations. PBE with DFT+D in CASTEP (red line) and PBEsol in CASTEP (green line) are applied to the solid of the $C_{10}H_{13}BO_{3}S$ compound, and B3LYP in Gaussian (blue line) is applied on the molecule of the $C_{10}H_{13}BO_{3}S$ compound. Note that the value of 5.27 ppm that appears in the experimental resonance is the one corresponding to the one impurity.

Figure 4 illustrates the magnetic resonance spectrum of the $C_{10}H_{13}BO_{3}S$ compound with experimental data and theoretical results. Although the model in CASTEP is applied to a solid (red and green lines), the experimental resonance spectrum is from a solution of the compound since the compound is dissolved in chloroform. In fact, the difference can be noticed in the Gaussian calculations (blue line), where the atoms of H7A and H8A have the same value, and the calculations made with CASTEP (on solids, periodic lattice Figure 2b) where the values H7A and H8A differ slightly from the experimental spectrum. It is also shown how the H3A and H4A hydrogen atoms of the thiophene ring exhibit less shielding, which means that the electrons are shifted, and their nuclei are less protected due to the inductive effect of electron-withdrawing groups (acetyl and boronic acid moieties) and the electron delocalization of the π electrons [41]. It implies that they appear in the magnetic resonance spectrum at a higher field, followed by the dioxaborolane H7A and H8A hydrogen atoms, and at a lower field than the H3A and H4A of the thiophene ring.

**Figure 5:**
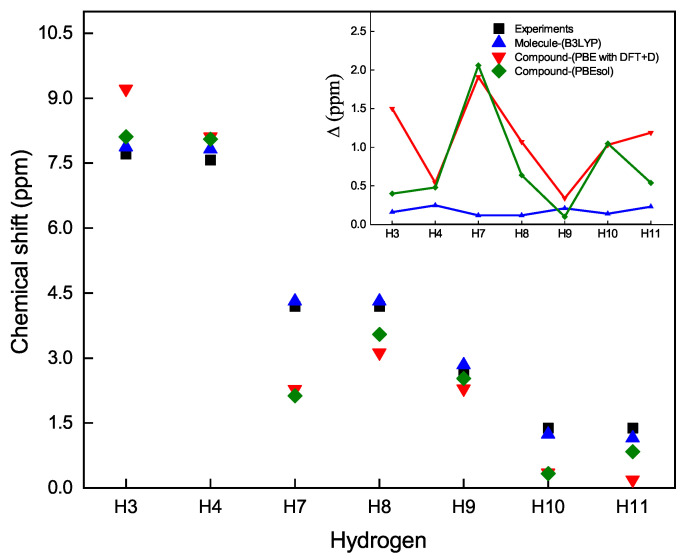
Chemical shift as a function of hydrogen atoms. Experimental values (black squares) of the chemical shift of hydrogen NMR-1H respect to the values calculated with different methods: B3LYP (blue triangle) in Gaussian on the molecule of the $C_{10}H_{13}BO_{3}S$ compound, PBE with DFT+D (red triangle), and PBEsol (diamond green) in CASTEP on the periodic lattice of the $C_{10}H_{13}BO_{3}S$ compound. Inset: Deviation of the theoretical values with respect to the experimental data, $\Delta =|CS_{th}-CS_{ex}|$, as a function of the hydrogen atoms.

An appropriate approach to show the chemical shifts of the hydrogen atoms in the $C_{10}H_{13}BO_{3}S$ compound is provided in Figure 5. In this figure, one can observe how far or close the theoretical results from the experimental data are. The inset of Figure 5 shows the deviation of the theoretical values from the experimental of the chemical shifts, $\Delta =|CS_{th}-CS_{ex}|$. In general, we observe that the computational model that best approximates the experimental calculations is that of the Gaussian software on the boronate ester molecule in solution.

On the other hand, we remark that the number of wave vectors *k* plays a crucial role in the calculation of nuclear magnetic resonance. For even greater accuracy, we should increase the number of wave vectors *k*, which would facilitate a better reading of the theoretical data with respect to the experimental data.

**Figure 6:**
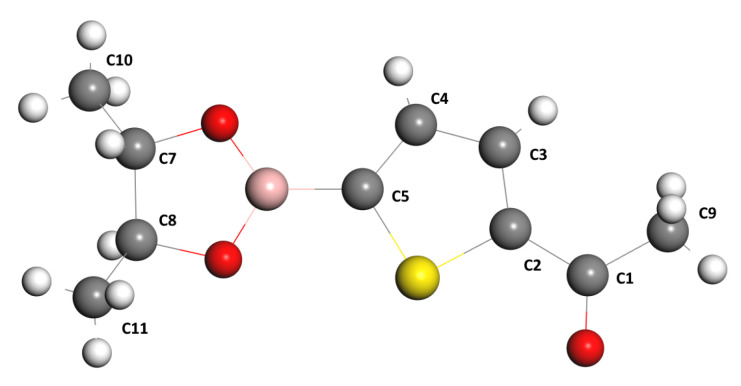
Label of the carbon atoms of the structure of the $C_{10}H_{13}BO_{3}S$ compound.

**Figure 7:**
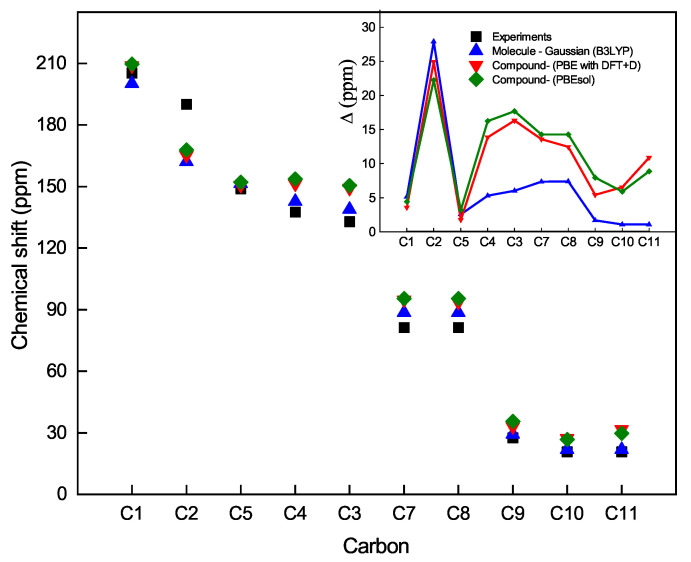
Experimental values (black squares) of the chemical shift of Carbon NMR-13C with respect to the values calculated with different methods; B3LYP (blue triangle) in Gaussian on the molecule of boronate ester, PBE with DFT+D (red triangle) and PBEsol (diamond green) in CASTEP on the periodic lattice of the $C_{10}H_{13}BO_{3}S$ compound. Inset: Deviation of the theoretical values respect to the experimental data, $\Delta =|CS_{th}-CS_{ex}|$, as a function of the Carbon atoms.

Now, let us analyze the chemical shift as a function of the carbon atoms from the NMR spectrum. For this reason, let us label the carbon atoms in Figure 6. Figure 7 shows the chemical shift as a function of the carbon atoms for both theories with the three methods mentioned above and experimental data. We observe that the carbon atom C1 is less shielded. Its electrons are shifted due to the inductive effect of the oxygen O1 atom, and accordingly, the nucleus of C1 is less protected, followed by the C2-5 carbon atoms of the thiophen ring at a higher field. The C7-8 atoms and the C9 atom of the acetyl moiety are more protected than the C1 atom or less exposed to the inductive effect of oxygen atom O1. If we compare the bond lengths C7-O2 (1.465 Å, optimized length) and C8-O3 (1.461 Å, optimized length) with the double bond length C1-O1 (1.246 Å, optimized length), it can be appreciated that the C1 atom of the acetyl moiety is less shielded and, therefore, its chemical shift appears at a lower field than the C9 atom of the methyl moiety. Finally, the C10-11 atoms of the methyl groups are more shielded, and consequently, their chemical shifts appear at a higher field. Moreover, the inset of Figure 7 shows the comparison between the theoretical and experimental chemical shifts that can be observed for the carbon atoms of the $C_{10}H_{13}BO_{3}S$ compound. Similarly, it can be seen that the same trend of the spectrum of the hydrogen atom is repeated, where the model that best approximates the experimental calculations is the Gaussian 09 on the boronate ester molecule in solution, which the CASTEP on the solid structure of the boronate ester.

## 5. Conclusions

Computational studies of the $C_{10}H_{13}BO_{3}S$ compound, such as NMR, electronic band structure, and density of state, have yet to be commonly done. In this work, we propose the characterization by applying the GIPAW method in CASTEP, which works on a periodic lattice structure, and the B3LYP method with the Gaussian, which works on molecules. A brief band structure study was carried out in order to visualize its electronic distribution, its density of state, and finally, the NMR study.

The calculations of the reference shielding $Si{\left(CH_{3}\right)}_{4}$ are compared with the data reported in the literature, obtaining a good approximation. This data can be used as a reference for the chemical shifts in the boronate esters. The 1-(5-(4,5-dimetil-1,3,2-dioxoborolan-2iltiofen-2-il) etanona was obtained from the reaction of the 5-acetyl-2-thienylboronic acid with 2,3-butanediol, where the results of their experimental resonances are also in agreement with the theoretical approach. Consequently, the chemical shifts of the ${}^{1}$H hydrogen atoms, the ${}^{13}$C carbon atoms and the ${}^{11}$B boron atoms, as well as, the the infrared spectroscopy.

Our study is expected to help guide future research, identifying conditions and methods that more accurately account for the properties of different molecules of interest to materials science.

## Figures and Tables

**Table 1:** Lattice parameters of the unitary cell of the $C_{10}H_{13}BO_{3}S$ structure.

Characteristic	Experimental [35]	Optimized Lattice Structure	
Empiric formula	$C_{10}H_{13}BO_{3}S$	-	-
Spacial group	P21/C(monoclinic)	PBE-GGA	PBEsol-GGA
Lattice parameters	a=6.038 Å	a=6.015 Å	a=6.046 Å
	b=10.286 Å	b=10.371 Å	b=10.303 Å
	c=20.096 Å	c=19.498 Å	c=20.643 Å
Lattice angles	$\alpha =90^{\circ }$	$\alpha =90^{\circ }$	$\alpha =90^{\circ }$
	$\beta =103.39^{\circ }$	$\beta =95.089^{\circ }$	$\beta =94.90^{\circ }$
	$\gamma =90^{\circ }$	$\gamma =90^{\circ }$	$\gamma =90^{\circ }$

**Table 2:** Optimized bond lengths C-H of the $C_{10}H_{13}BO_{3}S$ compound.

Bonds (All Lengths in Å)	Experimental Bond Lengths [35]	Optimization (CASTEP-PBE)	Optimization (CASTEP-PBEsol)	Optimization (Gaussian-B3LYP)
C3–H3A	0.930	1.088	1.097	1.081
C4–H4A	0.930	1.088	1.097	1.081
C7–H7A	0.980	1.100	1.110	1.094
C8–H8A	0.980	1.100	1.109	1.094
C9–H9	0.960(1)	1.097(2)	1.103	1.091
C10–H10	0.960	1.097(1)	1.105	1.092
C11–H11	0.960(1)	1.097(1)	1.105	1.092

**Table 3:** Optimized bond lengths C-C of the $C_{10}H_{13}BO_{3}S$ compound.

Bonds (All Lengths in Å)	Experimental Bond Lengths [35]	Optimization (CASTEP-PBE)	Optimization (CASTEP-PBEsol)	Optimization (Gaussian-B3LYP)
C1–C2	1.466	1.466	1.327	1.469
C2–C3	1.362	1.389	1.391	1.379
C3–C4	1.401	1.411	1.412	1.409
C4–C5	1.373	1.391	1.394	1.380
C1–C9	1.496	1.509	1.507	1.512
C7–C8	1.532	1.558	1.558	1.549
C8–C11	1.484	1.514	1.514	1.514
C7–C10	1.510	1.516	1.516	1.514

**Table 4:** Optimized bond lengths of the $Si{\left(CH_{3}\right)}_{4}$.

Bonds (All Lengths in Å)	Experimental Bond Lengths [35]	Optimization (CASTEP-PBE)	Optimization (CASTEP-PBEsol)	Optimization (Gaussian-B3LYP)
C–H	1.140	1.102(2)	1.106	1.084
C–Si	1.940	1.887	1.886	1.886

**Table 5:** Optimized bond lengths of the $BF_{3}OEt_{2}$.

Bonds (All Lengths in Å)	Experimental Bond Lengths [38]	Optimization (CASTEP-PBE)	Optimization (CASTEP-PBEsol)	Optimization (Gaussian-B3LYP)
C–H	1.070	1.070(2)	1.101	1.090
O–B	1.540	1.540	1.682	1.611
F–B	1.460	1.460	1.365	1.373

**Table 6:** Chemical shielding of ${}^{1}$H, ${}^{13}$C for $Si{\left(CH_{3}\right)}_{4}$ and ${}^{11}$B for $BF_{3}OEt_{2}$.

Elements σ (ppm)	Method	${}^{1}$H	${}^{13}$C	${}^{11}$B
	CASTEP GIPAW PBE (DFT-D)	30.576	179.400	88.080
(in this work)	CASTEP GIPAW PBEsol	29.900	181.230	91.370
	Gaussian-09 B3LYP	31.930	183.770	99.770
[39]	Gaussian-94 B3LYP/6-311	31.275	179.150	-
[40]	Gaussian-94 B3LYP/6-311	31.880	182.470	-
[26]	Q-Express GIPAW	30.800	179.330	-

**Figure A1:**
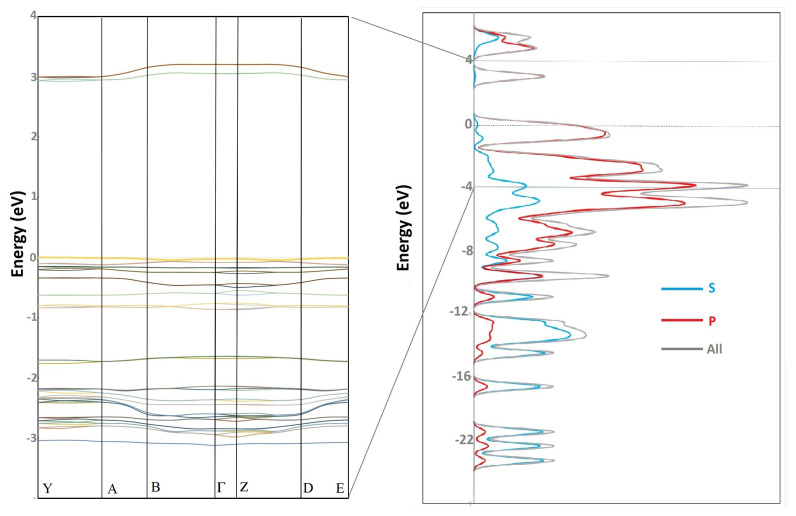
Band structure of the composite, indicating a band gap of 2.94 eV between the high symmetry points of the Y-A reciprocal lattice, the highest energy band below the Fermi level indicates 0 eV.

## Data Availability

The data presented in this study are available on request from the corresponding author.

## Appendix A. Band Structure and Density of the State

The band structures show how the energies depend on the wave vector k along points of high symmetry in the reciprocal lattice. These graphs provide a tool for analyzing the electronic structure of a material. The band structure graphs also deduce the energy gap since they correspond to the energy difference between two states at points of high symmetry. That is, it allows us to identify whether the material is conductive.

To calculate the band structure and density of state, we must first consider the optimization performed with the PBE exchange and correlation functional with DFT+D given in Table 1, then from these parameters, the same functional with a set of plane wave and augmented wave projector functions, a cutoff energy of 500 eV, a Monkhorst-Pack scheme to generate points k in a lattice 11×6×3. Then, we choose the points of high symmetry to be plotted in the band structure, the unit vectors of the direct lattice for construction of the reciprocal lattice, and the points of high symmetry are in Table A1.

**Table A1:** Conventional lattice vectors and high symmetry points of the reciprocal of the $C_{10}H_{13}BO_{3}S$ compound.

Real Space Vectors	High Symmetry Points of the Reciprocal Space
A1=(a,0,0)	Y=(0.00,0.50,0.00)
A2=(0,b,0)	A=(−0.50,0.50,0.00)
$A3=(c_{x},0,c_{z})$	B=(−0.50,0.00,0.00)
	Γ=(0.00,0.00,0.00)
$c_{x}=c\cdot Cos\left(\beta \right)$	Z=(0.00,0.00,0.50)
$c_{z}=c\cdot Sin\left(\beta \right)$	D=(−0.50,0.00,0.50)
	B=(−0.50,0.50,0.50)

Figure A1 shows the band structure and the state density of the $C_{10}H_{13}BO_{3}S$ compound, in the range of −4 eV to 4 eV, with an energy gap of 2.94 eV, indicating that it is an insulating or semiconducting material. The major contribution to the energy below the Fermi level is due to *S* and *P* orbitals. Above the Fermi level, the contribution is more from *P* orbitals.
